# Comparative Phytochemical Profiling and* In Vitro* Antioxidant Activity of Extracts from Raw Materials, Tissue-Cultured Plants, and Callus of* Oroxylum indicum* (L.) Vent.

**DOI:** 10.1155/2017/6853212

**Published:** 2017-10-31

**Authors:** Piyanuch Rojsanga, Somnuk Bunsupa, Adelheid H. Brantner, Pongtip Sithisarn

**Affiliations:** ^1^Department of Pharmaceutical Chemistry, Faculty of Pharmacy, Mahidol University, Bangkok 10400, Thailand; ^2^Department of Pharmacognosy, Faculty of Pharmacy, Mahidol University, Bangkok 10400, Thailand; ^3^Institute of Pharmaceutical Sciences, Pharmacognosy, University of Graz, 8010 Graz, Austria

## Abstract

Extracts from raw materials from different plant parts, tissue-cultured plants, and callus cultures of* Oroxylum indicum* were analyzed for* in vitro* antioxidant activities determined by DPPH radical scavenging assay and evaluated for phytochemical profiles by TLC and LC-MS methods. The results were analyzed by principal component analysis (PCA) to evaluate the similarity. Stalk, pedicel, flower, seed, and whole fruit and callus extracts promoted strong antioxidant activity with high total phenolic and total flavonoid contents. The main phytochemicals found in extracts were baicalin, baicalein, and chrysin. Baicalein and baicalin promoted strong antioxidant effects and existed in most extracts while chrysin, which promoted very low antioxidant activity, was a major flavonoid in the leaves and tissue-cultured plants. From PCA analysis by total phenolic and total flavonoid contents, four main clusters including callus and tissue-cultured plant groups from different growth stages, flower group, and whole fruit and leaf group could be organized. When the results were analyzed by PCA using antioxidant activity with total phenolic or total flavonoid contents, all* O. indicum* samples could be grouped together except the extracts from the root of tissue-cultured plants which separated from the rest due to their low phytochemical contents and weak antioxidant activities.

## 1. Introduction

*Oroxylum indicum* (L.) Vent. is a medium-sized, deciduous tree of the Bignoniaceae family. The tree has very large pinnate compound leaves. The outside petals are reddish purple with the pale yellow inside. Fruits are flat capsules, broad and sword shaped [1]. The seeds are numerous, flat-like papery wings [2]. Mature fruit is acrid and sweet, which promotes antihelminthic and stomachic effects [3]. The seeds have been used as purgative while the seed paste is applied to the throat for quick relief of tonsil pain [1, 2]. Our previous study reported the* in vitro* antibacterial and antioxidant activities of* O. indicum* fruit extracts [4]. Furthermore, the young fruits and young flowers of this plant are popularly consumed as vegetable in the North of Thailand while the mature seeds are one composition in a traditional multiherb drink for the treatment of aphthous ulcer and sore throat. Some phytochemicals were reported from different parts of* O. indicum* such as flavonoids, anthraquinones, alkaloids, saponins, and fatty acids [4–7]. Despite every part of* O. indicum* being useful for both nutritional and medicinal applications, however, the source of* O. indicum* raw materials seems to be limited. Moreover, it takes some times for this plant to fully grow as a tree for its appropriate uses. Therefore, finding the methods to increase and develop new sources of plant raw material would be beneficial in the future. Plant tissue culture technique is widely used in the conservation and utilization of rare and endangered medicinal plants due to its remarkable ability of quickly increasing their biomass [8, 9].

To our knowledge, no documents have ever been published about phytochemical profiles and antioxidant activities of the raw materials from different plant parts, tissue-cultured plants, and callus by chromatographic and spectrophotometric methods. This study provides the information of* in vitro* free radical scavenging effects tested by the DPPH assay, TLC and LC-MS fingerprints, and total phenolic and total flavonoid contents of extracts from* O. indicum* from different sources including flowers, leaves and fruits raw materials, tissue-cultured plants from different growth stages, and callus. The obtained results were also analyzed for similarity by principal component analysis (PCA).

## 2. Materials and Methods

### 2.1. Materials

#### 2.1.1. Plant Material Preparations

*(1) Raw Materials from Different Plant Parts.* The flowers (3 growth stages), pedicels, leaves, and stalks of* O. indicum* were collected from Bangkok province. The fruits for preparation of fruit and callus extracts were purchased from Chiang Rai province, while the seeds for preparation of tissue-cultures plants were purchased from Pattani province, Thailand, in 2016. Plant samples were identified by Assistant Professor Dr. Pongtip Sithisarn, Department of Pharmacognosy, Faculty of Pharmacy, Mahidol University, Thailand. The plant samples were separately cleaned and dried in a hot air oven at 60°C and then ground using an electric mill. 

*(2) Tissue-Cultured Plants (In Vitro Plants).* The seeds of* O. indicum* were cleaned using detergent and tap water flow for 15 min. Then they were sterilized by soaking in 70% ethanol for 30 sec followed by soaking in 1% sodium hypochlorite for 7 min. The sterilized seeds were washed by shaking in deionized water for 1 min, repeated for 5 times. Then the wings of the seeds were removed and the seeds were placed on sterilized MS (Murashige and Skoog) media (pH 5.8, 3% sucrose and 0.8% agar) [10]. Each media bottle contained 3 seeds of* O. indicum*. The bottles were kept in the dark until week 5 to induce germination and then they were moved to store under the photoperiod of 16/8 h (light/dark) until week 8. Plant samples were randomized using True Random Number Service software (Randomness and Integrity Services Ltd., Ireland) at days 3, 5, and 7 and then weekly from week 2 to week 8. Plant samples were cleaned and dried in a hot air oven at 60°C then ground using an electric mill. Plant samples from week 5 to week 8 were separated for the aerial and root parts before they were cleaned and dried.

*(3) Callus*. The seeds of* O. indicum* were separated from the fruits from Chiang Rai province and then they were washed with tap water for 15 min before sterilization with 70% ethanol for 1 minute. After that, the seeds were further sterile with 1% sodium hypochlorite for 8 minutes and then washed with sterile distilled water (5 times) to remove the remaining sterilization reagents. After the sterilization process, the wing part of the seeds was cut with sterile scissors in a laminar cabinet and the seeds then were placed in MS media supplemented with 3% sucrose and 0.8% agar before being stored in a dark place to induce the germination. After seven days, the germinated* in vitro* plants were moved to store under the photoperiod of 16/8 h (light/dark).

**Figure 1:**
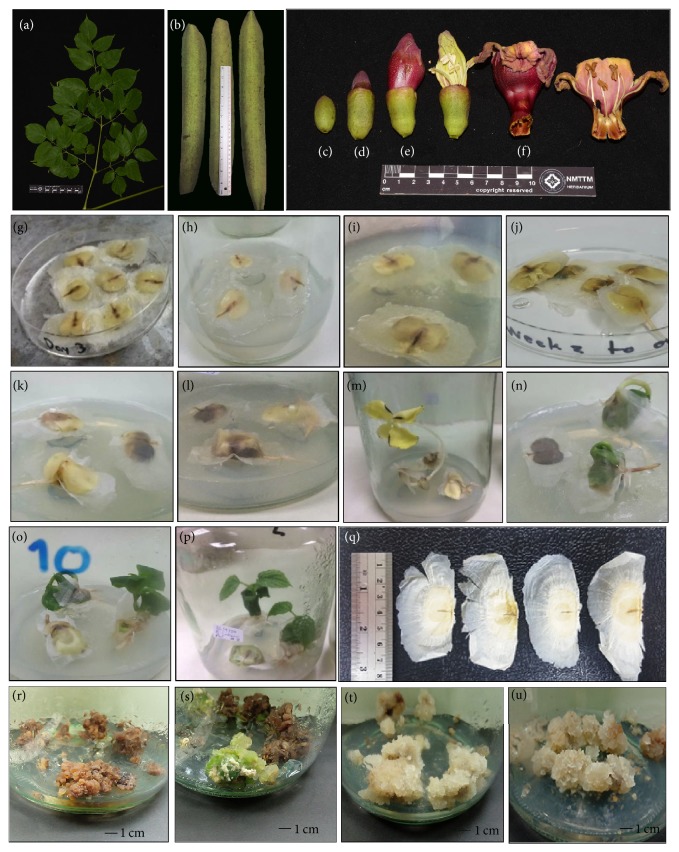
Physical characteristics of* Oroxylum indicum* samples. (a) = leaves, (b) = whole fruit, (c) = flower (stage 1), (d) = flower (stage 2), (e) = flower (stage 3), (f) = flower (bloom), (g) = tissue-cultured plant (day 3), (h) = tissue-cultured plant (day 5), (i) = tissue-cultured plant (day 7), (j) = tissue-cultured plant (week 2), (k) = tissue-cultured plant (week 3), (l) = tissue-cultured plant (week 4), (m) = tissue-cultured plant (week 5), (n) = tissue-cultured plant (week 6), (o) = tissue-cultured plant (week 7), (p) = tissue-cultured plant (week 8), (q) = mature seed, (r) = brown callus, (s) = green callus, (t) = white callus, and (u) = yellow callus.

The* in vitro* plants were collected after 8 weeks of cultivation and excised to explants (2-3 cm long/piece). The explants were inoculated in MS media supplemented with 3% sucrose, 0.8% agar, 2 mg/L of 2,4-dichlorophenoxy acetic acid (2,4-D), and 1 mg/l of 6-benzyladenine (BA). After 21 days, the induced callus was cut from the explants and cultured into the mentioned media and then subcultured every 21 days for three more times. After that, the callus was cultured to 28 days before being collected, dried, and then grinded to powder. The powdered callus was subjected for the further extraction process.  Figure 1 shows physical characteristics of* O. indicum* samples.

### 2.2. Methods

#### 2.2.1. Plant Extract Preparation

Each* O. indicum* sample was separately extracted by maceration using 95% ethanol (plant : solvent ratio 1 : 20 w/v) with continuous shaking for 6 h; then it was left for 12 h [4]. After that, the solution was filtered. Each extraction was repeated three times. The extracts were then combined and dried using a water bath.

#### 2.2.2. Determination of Antioxidant Activity by DPPH Scavenging Assay

The free radical scavenging activity of extracts from* O. indicum* sample was investigated using the 1,1-diphenyl-2-picrylhydrazyl (DPPH) radical scavenging method [11]. A total of 100 *μ*L of the extract or standard was added to 100 *μ*L of DPPH in a methanolic solution (152 *μ*M). After staying at room temperature for 15 min, the absorbance of each solution was determined at 515 nm using a microplate reader (Tecan, USA). The percentage of inhibition was calculated. Then the EC_50_ value of the samples required for 50% scavenging of the DPPH free radical was determined. Each determination was done in triplicate, and the average EC_50_ value was calculated.

#### 2.2.3. Phytochemical Studies

*(1) Determination of Total Phenolic Content Using Folin-Ciocalteu Method.* Plant extract solutions (25 *μ*L) were oxidized with Folin-Ciocalteu reagent (25 *μ*L) in 96-well plate. 75 *μ*L of distilled water and 100 *μ*L of 20% sodium carbonate solution were added. The absorbance of the resulting blue colored solution was measured at 765 nm after 60 min using a Microplate Reader (Tecan, USA) [12]. Each sample was done in triplicate. Total phenolic content was calculated from the standard curve of gallic acid and was expressed as g gallic acid equivalent in 100 g extract (g% GAE).

*(2) Determination of the Total Flavonoid Content.* Plant sample solutions (100 *μ*L) were separately reacted with a 2% aluminium chloride solution in the same volume. The absorbance was measured at 415 nm after 10 min using a Microplate Reader (Tecan, USA) [13]. Flavonoid content was calculated with the standard curve of quercetin and was expressed as g quercetin equivalent in 100 mg of plant extracts (g% QE). 

*(3) Thin Layer Chromatographic (TLC) Fingerprints. O. indicum* extracts were spotted on precoated silica gel 60 GF254 TLC plates. The plates were developed in two solvent systems, which were solvent system A: ethyl acetate : glacial acetic acid : formic acid : hexane (5 : 1 : 1 : 5, *v*/*v*/*v*/*v*) and solvent system B: ethyl acetate : toluene : formic acid (25 : 25 : 7.5, *v*/*v*/*v*). The developing distance was 85 mm. After being removed from the developing chamber, the TLC plates were air dried in a fume hood and examined under UV light at wavelengths of 254 and 366 nm and under UV light at a wavelength of 366 nm after spraying with natural products/polyethylene glycol (NP/PEG) reagent [4]. The TLC fingerprints of* O. indicum* extracts were recorded. 

*(4) Ultrahigh Performance Liquid Chromatographic-Mass Spectrometry (LC-MS) Fingerprints.* LC-MS analysis of all extracts from* O. indicum* was conducted using the method applied from Krüger and Ganzera [14] with Ultimate 3000 machine equipped with photodiode array and mass spectrometry detectors. A Kinetex C18 column (2.10 mm i.d. × 10 cm, 2.6 *μ*m) was used for quantitative analysis. Gradient elution was performed with 0.1% formic acid in water (solvent A) and acetonitrile (solvent B) at a constant flow rate of 0.35 mL/min. The gradient program was adjusted from 30% to 90% B in 10 min and stayed at 90% B for 2 min. Then the gradient program was adjusted to 30% B and stayed at 30% B for 3 min. Column temperature was 30°C with an injection volume of 3 *μ*L. Injection concentrations of standard references and plant samples were 10 and 100 *μ*g/mL, respectively. UV detection was also performed at 280 nm.

#### 2.2.4. Principal Component Analysis (PCA)

Data sets of DPPH scavenging activities (EC_50_ values), total phenolic, and total flavonoid contents of extracts from different plant parts and growth stages of* O. indicum* were subjected to Principal Component Analysis (PCA) using Minitab® (Minitab Pty Ltd., Australia).

#### 2.2.5. Statistical Analysis

All data are reported as means ± standard deviation of triplicate analysis. Least significant difference was used to compare means (*p* < 0.05). All analyses were performed using SPSS for Windows, version 16.0 (SPSS Inc., USA).

## 3. Results and Discussion

### 3.1. Physical Characteristics of Tissue-Cultured Plants

The physical characteristics of tissue-cultured plants are shown in Figure 1. From the starting day to day 7 of growing, there was no apparent change in physical characteristics of* O. indicum* seeds (Figures 1(g)–1(i)). In weeks 2 and 3, the roots started to appear, elongate, and root into the media (Figures 1(j) and 1(k)). In week 4, the length of the roots was about 3–5 cm (Figure 1(l)). The stems of* O. indicum* developed out of the seeds in week 5 and the stem length was about 6–8 cm with the light yellow cotyledons (Figure 1(m)). After the tissue-cultured plants were placed under the photoperiod of 16/8 hr light/dark condition, the color of the cotyledons changed from light yellow to green in week 6 (Figure 1(n)). The leaves started to grow in week 7 and clearly developed in week 8 with the length of the leaves around 3 cm; the stem was strong with the root length around 10 cm (Figures 1(o) and 1(p)).

### 3.2. Physical Characteristics of* Oroxylum indicum* Callus

The 28-day calluses from the leaves of* O. indicum in vitro* plants (8 weeks old) in MS media supplemented with 3% sucrose, 0.8% agar, 2 mg/L of 2,4-D, and 1 mg/l of BA can be divided into 4 types of samples: brown, green, white, and yellow calluses as shown in Figures 1(r)–1(u), respectively.

### 3.3. Physical Characteristics of* Oroxylum indicum* Extracts

The extracts from various parts of* O. indicum* (OI) including the seeds (OIMS), whole fruits (OIWF), flower stage 1 (OIFL1), flower stage 2 (OIFL2), flower stage 3 (OIFL3), flower stage 4 (OIFL4), leaves (OIL), pedicel (OIPC), stalk (OIS), tissue-cultured plant day 3 (OITPd3), tissue-cultured plant day 5 (OITPd5), tissue-cultured plant day 7 (OITPd7), tissue-cultured plant week 2 (OITPw2), tissue-cultured plant week 3 (OITPw3), tissue-cultured plant week 4 (OITPw4), the root of tissue-cultured plant week 5 (OITPw5R), the shoot of tissue-cultured plant week 5 (OITPw5S), the root of tissue-cultured plant week 6 (OITPw6R), the shoot of tissue-cultured plant week 6 (OITPw6S), the root of tissue-cultured plant week 7 (OITPw7R), the shoot of tissue-cultured plant week 7 (OITPw7S), the root of tissue-cultured plant week 8 (OITPw8R), the shoot of tissue-cultured plant week 8 (OITPw8S), brown callus (OICB), green callus (OICG), yellow callus (OICY), and white callus (OICW) appeared as brown semisolid matters with the yields shown in Table 1.

### 3.4. Determination of the Antioxidant Activity of* Oroxylum indicum* Extracts

The free radical scavenging activity of* O. indicum* extracts and of standard ascorbic acid, baicalin (Pharmaceutical grade, TRC, Canada), baicalein (analytical reference grade, Sigma-Aldrich, USA), and chrysin (Pharmaceutical grade, TRC, Canada), was investigated using the DPPH radical scavenging method (Table 1). Ascorbic acid, baicalein, and baicalin promoted strong antioxidant activities (EC_50_ values of 5.10, 8.98 and 32.49 *μ*g/mL, resp.) while chrysin showed low antioxidant effect (EC_50_ values higher than 500 *μ*g/mL). Extracts from* O. indicum* exhibited antioxidant activity varying from weak to strong effects. The mature seed (OIMS) and whole fruit (OIWF) extracts showed similar good antioxidant effects (EC_50_ values of 73.81 and 65.89 *μ*g/mL, resp.).* Oroxylum indicum* flower extracts indicated moderate to high antioxidant activity (EC_50_ values in the range of 26 to 85 *μ*g/mL). The bud (OIFL1) and the blossom flower (flower stage 4, OIFL4) extracts revealed very strong antioxidant effects (EC_50_ values of 48.14 and 26.04 *μ*g/mL, resp.) while the bud stage 2 (OIFL2) and bud stage 3 (OIFL3) demonstrated a lower activity (EC_50_ values of 71.69 and 84.64 *μ*g/mL, resp.). Pedicel (OIPC), stalk (OIS), and leaf (OIL) extracts also promoted strong antioxidant effects (EC_50_ values in the range of 17 to 65 *μ*g/mL). Most of tissue-cultured plant extracts exhibited weak DPPH scavenging effects (EC_50_ values in the range of 68 to 610 *μ*g/mL). In the early stages of the tissue culture of day 3–week 2 (OITPd3–OITPw2), the extracts showed moderate to low antioxidant effects (EC_50_ values in the range of 68 to 165 *μ*g/mL), which decreased significantly following the growth stages of the tissue-cultured plants. During week 3–week 7, the tissue-cultured plant extracts (OITPw3–OITPw7) exhibited low antioxidant activities (EC_50_ > 100 *μ*g/mL). The root extracts showed weaker antioxidant effects than those from the shoots. However, the antioxidant activity of the shoot extract from tissue-cultured plant at week 8 (OITPw8S) was noticeably risen again to the moderate level; this may suggest the change of biosynthesis of the secondary metabolite in* O. indicum* plant culture at this growth stage. Interestingly, all extracts from different colors of callus cultures (OIC) of* O. indicum* exhibited strong DPPH scavenging effects. The brown callus culture (OICB) significantly showed the highest antioxidant effect.

### 3.5. Phytochemical Study of* Oroxylum indicum* Extracts

#### 3.5.1. Determination of Total Phenolic and Total Flavonoid Contents by Spectrophotometric Techniques

Extract from various parts of* O. indicum* were quantitatively analyzed for total phenolic and total flavonoid contents using Folin-Ciocalteu and aluminium chloride methods, respectively. Phenolic and flavonoid contents were significantly responsible for the DPPH scavenging effects of* O. indicum* extracts. As shown in Table 1, extracts that promoted strong antioxidant effects including flower stage 1 (OIFL1), mature seed (OIMS), whole fruit (OIWF), pedicel (OIPC), and leaf (OIL) extracts contained high amounts of total phenolic compounds and total flavonoids (higher than 4 g% GAE and 3 g% QE, resp.). Extracts from tissue-cultured plants in early stages (OITPd5, OITPd7, OITPw2, and OTPw3) and callus cultures (OICB, OICG, OICW, and OICY) promoted the similar trends of high total phenolic contents (higher than 5 g% GAE) with moderate amounts of total flavonoids (around 1-2 g% QE). The stalk extract (OIS) promoted the highest antioxidant activity. However, the total phenolic and total flavonoid contents in this extract are in moderate level (2 g% GAE and 3 g% QE, resp.) suggesting the presence of other phytochemicals that support the antioxidant effects such as tannin and ascorbic acid. The late stages of tissue-cultured plants (OITPw4–OITPw8) promoted extracts with low to moderate phenolic and flavonoid contents (1–4 g% GAE and cannot be detected −3 g% RE, resp.) corresponding to their weak antioxidant effects. The results suggest that in* O. indicum* tissue-cultured plants, phenolic compounds, especially flavonoids, are stored mainly in shoot, but the amounts of them are very low in the root (lower than 1 g% QE).

#### 3.5.2. TLC Fingerprints of* Oroxylum indicum* Extracts

**Figure 2:**
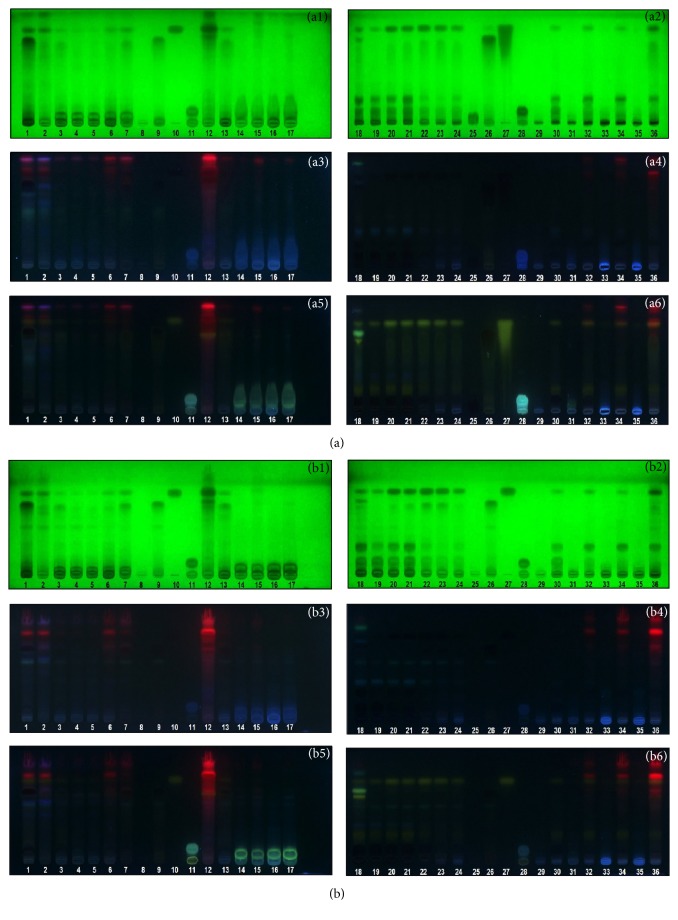
TLC chromatogram of* Oroxylum indicum* extracts; 1 = OIFL1, 2 = OIFL2, 3 = OIFL3, 4 = OIFL4, 5 = OIFL4, 6 = OIS, 7 = OIPC, 8 = baicalin, 9 = baicalein, 10 = chrysin, 11 = chlorogenic acid, 12 = OIL, 13 = OIWF, 14 = OICB, 15 = OICG, 16 = OICW, 17 = OICY, 18 = OIMS, 19 = OITPd3, 20 = OITPd5, 21 = OITPd7, 22 = OITPw2, 23 = OITPw3, 24 = OITPw4, 25 = baicalin, 26 = baicalein, 27 = chrysin, 28 = chlorogenic acid, 29 = OITPw5R, 30 = OITPw5S, 31 = OITPw6R, 32 = OITPw6S, 33 = OITPw7R, 34 = OITPw7S, 35 = OITPw8R, and 36 = OITPw8S, adsorbent: silica gel GF254. Solvent system: A = hexane-ethyl acetate-acetic acid-formic acid (5 : 5 : 1 : 1* v*/*v*/*v*/*v*), B = toluene-ethyl acetate-formic acid (25 : 25 : 7.5* v*/*v*/*v*), detection: 1 = UV 254 nm, 2 = UV 366 nm, and 3 = NP/PEG under UV 366 nm. Band identification system A: baicalin (Rf = 0.06), chlorogenic acid (Rf = 0.11), baicalein (Rf = 0.64), and chrysin (Rf = 0.71). Band identification system B: baicalin (Rf = 0.02), chlorogenic acid (Rf = 0.11), baicalein (Rf = 0.60), and chrysin (Rf = 0.70).

Thin layer chromatographic fingerprints of extracts from different plant parts and growth stages of* O. indicum* were phytochemically analyzed using 2 different solvent systems and detected under UV lights and NP/PEG spraying reagent. All extracts promoted specific chromatographic characteristics with the presence of phenolic and flavonoid compounds and extracts from the same plant parts promoted similar fingerprints as shown in Figure 2. Baicalein and chrysin (Rf 0.64 and 0.71 in solvent system A and 0.60 and 0.70 in solvent system B, resp.) showed the same trend in the extracts from the fruits, seeds, flowers, pedicels, and stalks of* O. indicum*. Their amounts are high in early stages of flowers (OIFL1 and OIFL2) but the amounts are decreasing in late stage flowers (OIFL3 and OIFL4), pedicel (OIPC) and stalk (OIS) extracts. Notably, in the leaf (OIL) and the tissue-cultured plant (OITP) extracts, chrysin was the major flavonoid. The results suggest that chrysin was biosynthesized and stored in the leaf part of* O. indicum* higher than other flavonoids. The amount of baicalin (Rf 0.06 and 0.02 in solvent system A and B, resp.) was quite low in all extracts. The extracts from callus cultures (OIC) slightly showed the bands corresponding baicalein and chrysin, but they mainly showed the chromatographic bands corresponded to phenolic compounds which appeared as bright blue bands after spraying with NP/PEG and detecting under UV 366 nm. The flower stage 1 (OIFL1), flower stage 4 (OIFL4), pedicel (OIPC), stalk (OIS), and brown callus (OICB) extracts, which promoted strong antioxidant effects, did not mainly show baicalein, baicalin, or chrysin bands while the tissue-cultured plant and leaf extracts contained mainly chrysin and promoted lower antioxidant effect. The results suggest that baicalein, chrysin, and baicalin are three main components in* O. indicum* and also exhibit an antioxidant effect. There should be other phenolics and flavonoids that are responsible for the antioxidant activity of this plant. This suggestion was supported by the TLC fingerprints that showed some other chromatographic bands reacting positive after spraying with NP/PEG reagent indicating that they should be phenolics and/or flavonoids.

#### 3.5.3. LC-MS Fingerprints of* Oroxylum indicum* Extracts

**Figure 3:**
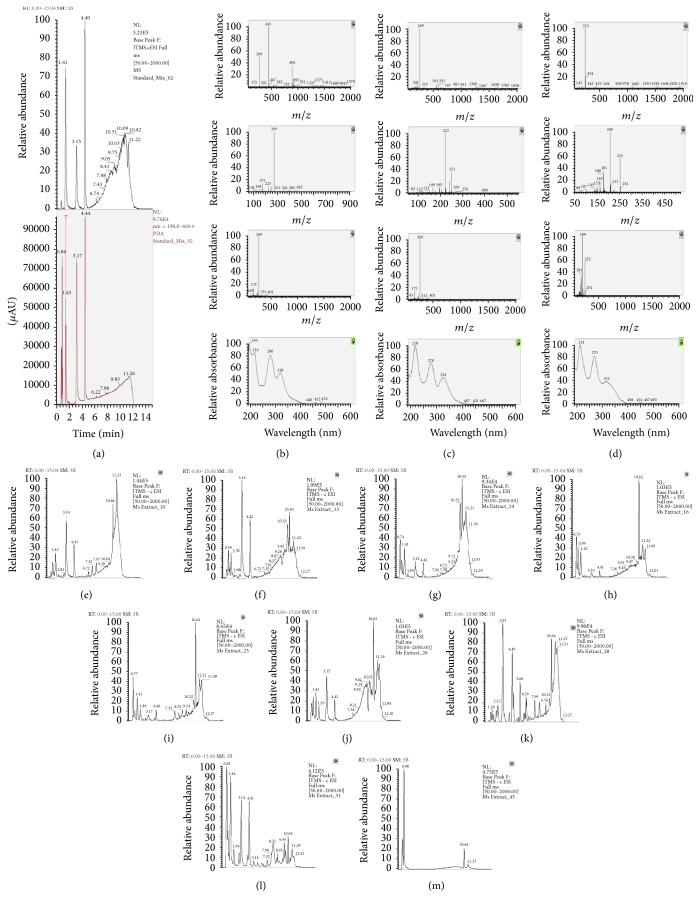
LC-MS chromatograms of* Oroxylum indicum* extracts; (a) = mixtures of reference compounds, (b) = mass spectra and UV spectra of baicalin, (c) = mass spectra and UV spectra of baicalein, (d) = mass spectra and UV spectra of chrysin, (e) = flower stage 1 extract, (f) = flower stage 2 extract, (g) = flower stage 3 extract, (h) = flower stage 4 extract, (i) = pedicel extract, (j) = stalk extract, (k) = leaf extract, (l) = whole fruit extract, and (m) = callus extract.

LC-MS was used to analyze and identify the peaks of the standards baicalin, baicalein, and chrysin. It was found that baicalin promoted the LC-MS peak at a retention time of 1.41 minutes with UV spectra at the maximum wavelength of 216, 280, and 318 nm and molecular mass (negative mode) of 445 *m*/*z*. Baicalein promoted the LC-MS peak at a retention time of 3.15 minutes with UV spectra at the maximum wavelength of 218, 278, and 324 nm and molecular mass (negative mode) of 269 *m*/*z*. Chrysin promoted the LC-MS peak at a retention time of 4.40 minutes with UV spectra at the maximum wavelength of 214, 270, and 315 nm and molecular mass (negative mode) of 253 *m*/*z*. Different plant parts and growth stages of* O. indicum* promoted different LC-MS chromatograms. The content of baicalin is quite stable in all flower extracts (estimated by manual observation of peak height and peak area). Baicalein and chrysin contents showed the same trends of LC-MS peaks. They are high in younger stages flowers (young buds stages 1 and 2) and they seem to decrease when the flowers are getting older (bud stages 3 and 4 which are bloom flower). Bloom flower extract showed the lowest baicalein and chrysin contents. The overall LC-MS chromatogram profiles of the three major chemical constituents baicalin, baicalein, and chrysin of the pedicel extract from* O. indicum* are similar to the bloom flower extract (constant baicalin content and low amounts of baicalein and chrysin) while the content of these 3 flavonoids in the stalk extract is different. This extract contains the amount of baicalin similar to others; however, the amounts of baicalein and chrysin are higher than the yield of these two compounds in bud stage 3, bloom flower, and pedicels extracts but lower than those of bud stages 1 and 2 extracts. Moreover, there is another peak at RT 1.93 minutes with the molecular mass of 253. This compound could be a flavonoid glycoside. The LC-MS profile of the leaf extract from* O. indicum* is different from those of flower and whole fruit extract. The amounts of baicalin and baicalein are very low, but the amount of chrysin is high. There are other peaks at RT 1.96, 2.91 and 4.61 minutes with the molecular masses of 253, 284, and 268 *m*/*z*, respectively. These compounds could also be flavonoids. From LC-MS analysis, the amounts of baicalin, baicalein, and chrysin in the whole fruit extract are high, especially the amount of baicalin which is higher than in the other extracts. There is a peak at RT 1.96 minutes with the molecular masses of 253. This compound could be a flavonoid. The LC-MS chromatograms from extracts of 4 different colors* O. indicum* calluses showed very low intensity peaks at a concentration of 100 *μ*g/mL. The reason, therefore, might be different batches and ages of the calluses used in this analysis. They were different from the other calluses used in other experiments in this study. The LC-MS chromatograms of extracts from* O. indicum* and reference compounds are shown in Figure 3.

### 3.6. Principal Component Analysis (PCA) of Results from* Oroxylum indicum* Extracts

**Figure 4:**
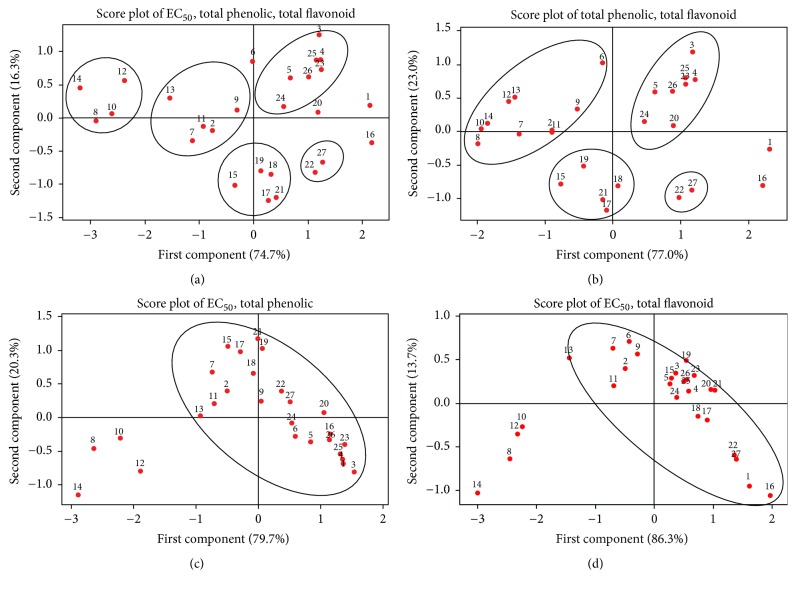
Score plot of principal component scores obtained from EC_50_ values from DPPH scavenging assay, total phenolic and total flavonoid contents of* O. indicum* extracts from various plant parts, and growth stages as shown in Table 1. 1 = OIMS, 2 = OITPd3, 3 = OITPd5, 4 = OITPd7, 5 = OITPw2, 6 = OITPw3, 7 = OITPw4, 8 = OITPw5R, 9 = OITPw5S, 10 = OITPw6R, 11 = OITPw6S, 12 = OITPw7R, 13 = OITPw7S, 14 = OITPw8R, 15 = OITPw8S, 16 = OIFL1, 17 = OIFL2, 18 = OIFL3, 19 = OIFL4, 20 = OIPC, 21 = OIS, 22 = OIL, 23 = OICB, 24 = OICG, 25 = OICW, 26 = OICY, and 27 = OIWF.

The PCA results were used to generate the projection plot (Figure 4) that provides a visual determination of the similarity among the* O. indicum* samples. The loading plot of* O. indicum* samples was obtained from their EC_50_ values from the DPPH scavenging assay and the total phenolic and the total flavonoid contents.  Figure 4(a) shows the overall plot of EC_50_ values from DPPH scavenging assay and total phenolic and total flavonoid contents of* O. indicum* samples. It was found that all samples scattered in disorder pattern. However, some samples could be grouped together such as extracts from callus including brown callus (23, OICB), green callus (24, OICG), white callus (25, OICW), yellow callus (26, OICY) extracts from young tissue-cultured plants 5 (OITPw2) and 3 (OITPd5), extracts from flowers 17 (OIFL2), 18 (OIFL3), 19 (OIFL4), and 21 (OIS), 15 (OITPw8S) and extracts from the root of tissue-cultured plant groups 8 (OITPw5R), 10 (OITPw6R), 12 (OITPw7R), and 14 (OITPw8R). Extracts from the leaf (22, OIL) and whole fruit (27, OIWF) showed some similarities and stayed close to each other in PCA score plots as shown in Figure 4. When the samples were grouped by total phenolic and total flavonoid contents as shown in Figure 4(b), 4 main clusters of* O. indicum* samples of callus and young tissue-cultured plant group, tissue-cultured plant group, flower group, and whole fruit and leaf group could be organized. It should be mentioned that the mature seed (1, OIMS) and flower stage 1 (16, OIFL1) are localized outside the area of other samples. Figures 4(c) and 4(d) suggest the similarity of* O. indicum* samples analyzed by EC_50_ values from DPPH scavenging assay and total phenolic content and EC_50_ values from DPPH scavenging assay and total flavonoid content, respectively. Similar trends were found in both plots; all* O. indicum* samples could be grouped together except the extracts from the root of tissue-cultured plants 8 (OITPw5R), 10 (OITPw6R), 12 (OITPw7R), and 14 (OITPw8R) which clearly separated from the rest of the samples.

This research is the first report of phytochemical profiles determined by TLC and LC-MS methods and* in vitro* antioxidant activities tested by the DPPH scavenging assay of extracts from different origins of* O. indicum* including raw materials from different plant parts (seeds, whole fruits, flowers, leaves, pedicels, and stalks), tissue-cultured plants from different growth stages, and callus cultures. Extracts from stalk, pedicel, bloom flower (flower stage 4), bud (flower stage 1), and calluses exhibited high antioxidant effects. Baicalin and baicalein which are present in most extracts showed high antioxidant effects while chrysin which is a major flavonoid in the leaves and tissue-cultured plants promoted very low antioxidant activity. This suggests that there are other compounds responsible for the antioxidant activities of* O. indicum*. Some flavones and their glycosides such as scutellarein, norwogonin, acacetin, hispidulin, oroxylin A, apigenin, and tetuin were previously reported in the leaves, seeds, stem bark, and root bark of* O. indicum* [15–20]. Flavonols, flavonoids with a hydroxyl group at the C-3 position, were reported to promote better antioxidant effect than other flavonoids without this functional group such as flavones [21]. Some flavonols previously reported in* O. indicum* including kaempferol and quercetin could also support the antioxidant effect of this plant [22]. There were studies about* in vitro* propagation by callus and shoot induction [23–26] and protoplast isolation [27] for the production of* O. indicum*. Auxin and cytokinin were reported as good growth regulators for* O. indicum* callus induction [28]. The recent study indicated that GA3 had a significant effect on seed germination of* O. indicum*. Moreover, total phenolic and total flavonoid contents were reported to be maximum in* in vitro* developed root compared to other* in vitro* plant parts [29]. Another report indicated the presences of baicalein and chrysin in callus extracts of* O. indicum* [30].

## 4. Conclusion

From all of the results, it could be summarized that the stalk, pedicel, flowers, seeds, fruits, and callus promoted the extract with high antioxidant activity and contained high total phenolic and total flavonoid contents with baicalin, baicalein, and chrysin as main ingredients found in these extracts. The shoot extracts from tissue-cultured plants also promoted some antioxidant effects but their contents of phenolics and flavonoids are quite low. Mature seeds, buds (flowers stage 1), blossoms (flowers stage 4), stalks, and leaves are interesting plant parts which should be further investigated for other active ingredients. Biotechnological studies should be provided for more research about callus and tissue-cultured plant material, especially the callus cultures which promoted a strong antioxidant effect and a high total phenolic content.

## Figures and Tables

**Table 1:** Total phenolic and total flavonoid contents and *in vitro* antioxidant activity of extracts from various parts and different growth stages of *O. indicum*.

Sample	Yield (% w/w)	Total phenolic content (g GAE/100 g extract)	Total flavonoid content (g QE/100 g extract)	Antioxidant activity (EC_50_, *μ*g/mL)
OIMS	17.54	7.31 ± 0.92^a,b^	5.31 ± 0.73^a,b^	73.81 ± 1.89^a^
OIWF	26.11	4.57 ± 0.45^c,d^	4.68 ± 0.12^b^	65.89 ± 5.48^a,b,c^
OIFL1	16.12	6.30 ± 0.46^a,e,f,g^	5.83 ± 0.28^a^	48.14 ± 0.92^d^
OIFL2	15.46	2.15 ± 0.24^h^	3.58 ± 0.17^c^	71.69 ± 3.01^a,e^
OIFL3	39.91	2.97 ± 0.31^i,j^	3.33 ± 0.10^c^	84.64 ± 2.49^f^
OIFL4	30.52	2.63 ± 0.33^i,k,l ^	2.41 ± 0.04^d^	26.04 ± 0.59^g^
OIL	22.02	4.10 ± 0.70^d,j,m^	4.59 ± 0.32^b^	63.21 ± 4.79^b,c^
OIPC	11.10	5.66 ± 0.45^f,g^	3.24 ± 0.18^c^	23.66 ± 1.47^g^
OIS	11.63	2.31 ± 0.22^h,k,l^	3.32 ± 0.10^c^	17.69 ± 1.55^h^
OITPd3	17.80	2.74 ± 0.59^h,I,j,k,l^	1.25 ± 0.08^e^	162.38 ± 6.34^i^
OITPd5	18.53	7.79 ± 1.02^a,b^	2.30 ± 0.21^d,f^	69.61 ± 2.67^a,c,e^
OITPd7	25.34	7.19 ± 0.85^a,b,e,g,n^	2.80 ± 0.17^g^	68.96 ± 0.36^c,e^
OITPw2	27.83	6.01 ± 0.29^b,f,n^	2.34 ± 0.12^d^	95.03 ± 1.06^j^
OITPw3	31.73	5.50 ± 0.46^e,f^	0.94 ± 0.24^e,h^	119.42 ± 3.08^k^
OITPw4	31.47	1.93 ± 0.54^h,k,l,o,p^	0.73 ± 0.08^h^	156.97 ± 4.16^i^
OITPw5R	10.38	0.74 ± 0.14^o^	0.19 ± 0.06^i^	500.98 ± 12.25^l^
OITPw5S	24.55	3.82 ± 0.18^d^	1.30 ± 0.11^e^	117.49 ± 19.81^j,k^
OITPw6R	18.90	1.17 ± 0.05^p,q^	Cannot be detected	435.21 ± 2.30^m^
OITPw6S	19.17	2.72 ± 0.35^i,k,l^	1.26 ± 0.11^e^	205.60 ± 2.25^n^
OITPw7R	16.11	2.43 ± 0.52^i,k,l^	Cannot be detected	454.36 ± 9.15^o^
OITPw7S	18.45	2.67 ± 0.14^i,j,l^	0.03 ± 0.00^j^	251.63 ± 2.95^p^
OITPw8R	19.10	1.44 ± 0.08^r^	Cannot be detected	607.57 ± 4.52^q^
OITPw8S	20.12	1.72 ± 0.40^h,q,r^	2.32 ± 0.27^d^	85.19 ± 2.39^f^
OICB	25.12	6.90 ± 0.45^a^	2.73 ± 0.03^g,k^	38.78 ± 1.32^r^
OICG	26.83	5.09 ± 1.00^d,e,f,g,m^	2.66 ± 0.07^g,k^	98.55 ± 1.92^j^
OICW	27.30	7.05 ± 0.90^a,b,f,g^	2.61 ± 0.06^f,g^	63.62 ± 3.32^b,c^
OICY	24.64	6.41 ± 0.91^a,b,f,g^	2.63 ± 0.03^f,g^	58.64 ± 2.59^b^
Ascorbic acid	—	—	—	5.10 ± 0.57
Chrysin	—	—	—	>500
Baicalein	—	—	—	8.98 ± 0.86
Baicalin	—	—	—	32.49 ± 12.52

Different letters in the same column are significantly different (*p* < 0.05); OIMS = OI seed extract, OIWF = OI whole fruit extract, OIFL1 = OI flower stage 1 extract, OIFL2 = OI flower stage 2 extract, OIFL3 = OI flower stage 3 extract, OIFL4 = OI flower stage 4 extract, OIL = OI leaf extract, OIPC = OI pedicel extract, OIS = OI stalk extract, OITPd3 = OI tissue-cultured plant day 3 extract, OITPd5 = OI tissue-cultured plant day 5 extract, OITPd7 = OI tissue-cultured plant day 7 extract, OITPw2 = OI tissue-cultured plant week 2 extract, OITPw3 = OI tissue-cultured plant week 3 extract, OITPw4 = OI tissue-cultured plant week 4 extract, OITPw5R = OI tissue-cultured plant week 5 root extract, OITPw5S = OI tissue-cultured plant week 5 shoot extract, OITPw6R = OI tissue-cultured plant week 6 root extract, OITPw6S = OI tissue-cultured plant week 6 shoot extract, OITPw7R = OI tissue-cultured plant week 7 root extract, OITPw7S = OI tissue-cultured plant week 7 shoot extract, OITPw8R = OI tissue-cultured plant week 8 root extract, OITPw8S = OI tissue-cultured plant week 8 shoot extract, OICB = OI brown callus extract, OICG = OI green callus extract, OICY = OI yellow callus extract, and OICW = OI white callus extract.
